# The influence of healthcare service quality on patients' satisfaction in urban areas: The case of Pakistan

**DOI:** 10.1016/j.heliyon.2024.e37506

**Published:** 2024-09-12

**Authors:** Abdul Rauf, Norhilmi Muhammad, Hamid Mahmood, Yuen Yee Yen

**Affiliations:** a Faculty of Business and Management, Universiti Sultan Zainal Abidin, Malaysia; b Faculty of General Studies and Advanced Education, Universiti Sultan Zainal Abidin, Malaysia; c Department of Management Sciences, TIMES Institute, Pakistan; d Faculty of Business, Multimedia University, Malaysia

**Keywords:** Satisfaction, Service quality, Urban medical care, Revisit intention, Basic health unit

## Abstract

An advanced hospital services is an imperative goal in the healthcare delivery process that might contribute to the patient's emotions and behavioral intention regarding the service experience. Therefore, current study aims to investigate the influence of healthcare service quality and patients' satisfaction with basic health unit (BHU) hospitals in Punjab, Pakistan. The study focuses on service quality, revisits intention and patients' satisfaction with the framework of the planned behaviour model. Quantitative research was conducted using a self-administered questionnaire from those patients who visited the same hospitals twice in a month. As result, the sampling strategy was simple random sampling (SRS) and sample size was (n = 469). The researchers used structural equation modelling (SEM) and AMOS to examine and evaluate the study hypotheses. The findings indicate that service quality increases patients' satisfaction and motivates them to revisit again. Service quality plays a crucial role in enhancing patients' intention to revisit the same hospitals and maintain their satisfaction level. The results provide valuable insights for medical marketing teams to promote and strengthen patients' intention to revisit to their medical care hospitals. Additionally, these findings may inform governments on how to maintain and improve medical facilities for their future patients. This research is among a limited number of studies that examine the predictive association between service quality, patients' satisfaction, and patients' tendency to revisits to government hospitals in Punjab, Pakistan.

## 1 Introduction

Basic healthcare units (BHU) in Pakistan serve as important access point to provide primary healthcare services to address the need of diverse and densely populated community. The BHUs in Pakistan run by government and designed to deliver crucial health services, including general outpatient services, maternal services, immunization, and child healthcare to extensive population to bridging gap in healthcare infrastructure [1]. As living conditions improve and access to information becomes more convenient, there is increasing revisit intention among patients to the same hospitals in some cases but not at all the times for the urban health unit [2]. Moreover, the significant surge in healthcare workers' and patients' satisfaction levels from the established healthcare centres is a problem for the modern urban planning management system [3]. Nowadays, patient satisfaction is attached to health service qualities and revisiting medical institutions for better and more reliable health facilities [4]. Medical institutions must match patients’ rising awareness and expectations and the growing number of medical services for customers to achieve sustainable urban planning management [5].

The healthcare industry is transitioning from a supplier-oriented approach to a customer satisfaction-oriented one for the current urban planning healthcare system [6]. Customer-centric marketing is a fundamental idea at the healthcare administration level, which seeks to discover and fulfil the demands of patients at the urban level [7]. Before this, the medical sector was controlled mainly by a supplier-centric market model, in which individuals would visit hospitals without obtaining medical care personalised to their specific needs [8]. Currently, the urban management system has primarily focused on patient healthcare satisfaction, as hospitals thrive by comprehending the varied requirements of medical consumers and delivering high-quality medical services to come again for treatment [9]. The fundamental objective of healthcare organizations is to provide high-quality services to medical customers and also their patients. The number of medical institutes in Pakistan is steadily rising, and the level of revisit intention and providing high-quality health care services to patients is increasing. Pakistan lies among those countries where people pay highest revenue out of pocket to get health care facilities [10]. The government of Pakistan is spending 2.88 % of their total GDP to finance healthcare system in the country [11]. Despite this, patients are switching for better healthcare service and only 30 % patients avail govt healthcare facility and 70 % percent get their treatment from private hospitals [12].

Therefore, to ensure the continued operation and success of healthcare institutions via gaining a competitive edge, it is necessary to implement high-quality health service for the patients and their satisfaction level to revisit again to the hospitals at the urban level. The extent scientific studies highlighted that reliability, responsiveness, and empathy were measured individually, and the current research has measured reliability, responsiveness, and empathy in combination and filled this gap. Similarly, there are very rare studies who measure revisit intention as an intervening factor between service quality and patient satisfaction. Furthermore, despite the importance of healthcare service quality at BHUs in Pakistan to understand success factors and operational challenges, there are very scare studies available who measure satisfaction and patients behavioral intention in this context. These insights are essential for healthcare administrators and policy makers to increase BHUs quality of care, thereby enhance overall health outcomes and meet healthcare needs of Pakistan urban population.

## 2 Theoretical framework of planned behavior

The theory of planned behaviour (TPB) was the fundamental lens of the study. Such as, Ajzen [13] states that attitude, perceived behavioural control and subjective norms mould individuals' behaviour and further shape behavioural intentions in the community. Different researchers used behavioural intentions and actions toward patients' response to quality and satisfaction regarding service [[14], [15], [16]]. Prior studies attempted to quantify the predisposition of revisit intention in terms of health service quality and their patient's behavioural intentions toward service satisfaction [17]; [18]. Some scholars have asserted that service quality maintains the revisit intention of the patients in urban geographical healthcare centres [19,20]. Consequently, Yuliana and Tham [21] stated that assessing revisit intentions in healthcare institutions is more important for the patient satisfaction with health facilities. Similarly, Lee and Kim [22] have posited that the healthcare system can gauge the disposition to endorse a hospital. Furthermore, the patient's intention is positively associated with satisfaction due to service quality. These factors help to improve the healthcare information among patients and serve as a valuable tool to convince their patients to revisit the same hospitals [23]; [24]. In light of this backdrop, several studies have endeavoured to ascertain the impact of service quality plans on patient satisfaction and returning to the same healthcare centres for taking healthcare facilities at the urban level [25,26]. However, their empirical findings suggested that healthcare service quality is associated with the outcome of patient satisfaction. As a result, much empirical data recommended that revisit intention significantly influences the patients' satisfaction and return to take facilities from the healthcare centres.

## 3 Hypothesis development

The literature review provides details of critical and scientific discussions toward health care service quality and patient satisfaction among urban healthcare units such as BHU through revisit intention.

### 3.1 Service quality and patient satisfaction

Health care facilities have to shift from a medical paradigm and adopt a more social perspective of healthcare. It is not only necessary to give exact diagnosis and cure to the patients but also, they also expect higher level of services [27,28]. Patient satisfaction is painstaking an ideal consequence among healthcare because it directly associated toward re-use of health-related services and healthcare facility success [29]. An extant study investigates the relationship between service quality and patient satisfaction in the health care sector in Saudi Arabia's province of Al-Baha and assessed the level of satisfaction of 400 inpatients with health services in Riyadh and concluded that the highest mean satisfaction score was in admission while the lowest in communication [30]. The favourable association between healthcare service quality and patients' satisfaction has been generally acknowledged for the betterment of the urban health unit [31]. Similarly, prior research in Turkey on satisfaction of patients find the issues that need perfection i.e., articulate the objective of quality enhancement, seldomly identify the development actions, and techniques of monitoring that exercised by the administration of hospitals, medical staff, operation staff and nursing [32]. Nevertheless, several authors focused on the precise nature of the predictive association and its ability to accurately forecast patient health satisfaction among healthcare facilities institutions, and it still needs to be clarified [33]; [34]. Thus, we hypothesized that.

H1 Service quality has an impact on patients' satisfaction.

### 3.2 Service quality and revisit intention

It is important to understand that managed healthcare organizations have a legal and moral responsibility to deliver safe and quality healthcare to the patient before managing operations [35]. Donabedian [36] argued that healthcare quality can be explained by the professional competency of the healthcare provider together with the perceptions of the patient about the rendered medical facility. The future positivistic research requires an awareness of external factors that affect the quality of treatment delivered. This is because patient satisfaction is influenced by personal expectations, and both service healthcare quality and revisiting the same hospital simultaneously to approach the healthcare services institutions again [37]; [38]. An extant study conducted in Korea highlighted that even number of medical institutions, their strategy aims to satisfy needs of patients i.e., quality medical services to gain a competitive edge in the healthcare institutions so as to continue to operate that be the cause of revisit and survival [8]. The degree of service quality refers toward hospital administrators to developed plans which include delivering quality services, and developing a pleasant experience so as to shape the patients’ behavior to nurture a long-term relationship that in turn, results to patients revisiting the same healthcare facility for their health care needs [39]; [40]. So, we hypothesized that.

H2 Service quality has an impact on revisit intention.

### 3.3 Revisit intention and patient satisfaction

According to Woo and Choi [8] there is an optimistic role of patients' satisfaction for triggering the intention to revisit. Basically, the services of service providers i.e., nurses, doctors, and supporting staff are the key factor that satisfy the patients which paved a way for intention to revisits. The perception of revisit intention derives from behavioral intentions [41] correspondingly, there is a dire need to understand the behavior of patients for service delivery which they wish accordingly [42]. By doing so, satisfaction of patients will easily obtain that be the cause of visit again and again. Lai et al. [40] stated that patient satisfaction is an essential outcome and primary objective of medical healthcare treatment. Previous studies have shown interest in patient satisfaction but needs more conceptualization regarding revisiting intention from the perspective of service qualities [43]; [44]. The lack of uniformity across many urban healthcare institutions and inconsistency regarding health facilities have generated concern among research scholars [45,46]. A recent study conducted in Indonesia explores the association among satisfaction of patients and their revisit intention toward the hospital to fulfil their needs of healthcare. It can also be highlighted that such consistent aspects provide momentous intuitions toward satisfaction among patients and their impact on the willingness of patients return [47]. So, we hypothesized that.

H3 Revisit intention has an impact on patients' satisfaction.

### 3.4 Mediating role of revisit intention

Service quality positively related with healthcare facilities and patient satisfaction that can influence whenever patient revisit to the same hospitals. Moreover, healthcare service quality research has shown a limited connection between patient satisfaction and the likelihood of returning to the same hospitals [48]. A number of researches prevailed on revisit intention in diverse context i.e., social media marketing perspective in North Cyprus context [49]; Kartarpur temple as religious perspective in Pakistani context [50]; tourism perspective in Malaysian context [51]; food perspective in Nanjing, China [52]; and behavioral perspective in Hong Kong context [53]. Almost all extant studies take revisit intention as a dependant construct but in present research, we use revisit intention as a mediating construct that be the novel injector as per researcher knowledge. Additionally, more research was need to investigate the intermediary healthcare function as a service quality and its predictive association with patient satisfaction. Thus, we hypothesized that.

H4 Revisit intention mediate the relationship between service quality and patients' satisfaction.

## 4 Conceptual framework

**Fig. 1:**
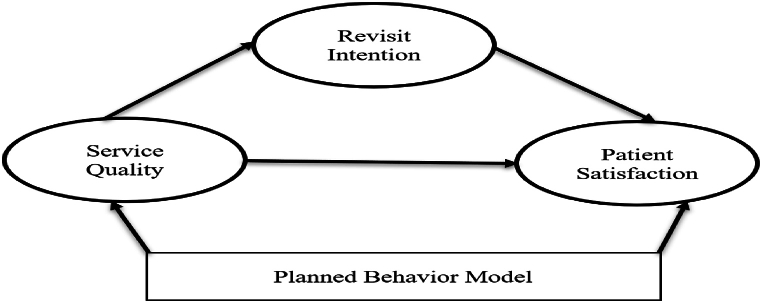
Conceptual framework.

Source: Behavioural Intentions Regarding Service Satisfaction [18].

The research paper applied a planned behaviour approach to test the revisit intention of the patient in the same hospitals in urban communities. Similarly, the service quality of the healthcare centres was an independent variable that could affect the patient's satisfaction level. The intervention of revisit intention was intervened between service quality and patient satisfaction level. The study used the assumption of planned behaviour and tested the proposed model, which is portrayed in Fig. 1.

## 5 Research Objectives

• To measure the effect of healthcare service quality on patient satisfaction level among urban citizens.
• To know the impact of healthcare service quality on revisit intention among urban citizens.
• To see the intervening role of revisit intention between healthcare service quality and patient satisfaction level among urban citizens.

## 6 Research design

This research received an ethical approval from the TIMES Institute, Office of Research, Innovation and Commercialization (ORIC), Multan, Pakistan, with reference no TI/ORIC/2023/86. The current research employs a quantitative methodology to assess the healthcare service quality and revisit intentions impact the level of patients' satisfaction among urban residents in developing nations. Additionally, the study examines the mediating effect of revisit intention in this proposed relationship. Tashakkori and Creswell [54] define quantitative research as studying objective reality. Multiple authors have described that quantitative research has established the characteristics of objectivity, which are universally applicable, inflexible, and persistent [55,56]. The use of quantitate approach in current study justify its ability to provide objective and generalizable results through measurement and statistical analysis. This approach also ensures replicability and precision, support testing of hypothesis, and simplifies data collection and analysis. The current study also enhances the methodological rigor and contributes in the body of knowledge by adapting validate instruments, ensuring finding reliable and widely applicable. The research was conducted in BHU hospitals of Sahiwal, Okara, and Bahawalpur, Punjab, Pakistan. The research focused on analysing patients as the primary unit of analysis. The research focused on analysing those patients who have revisited to these BHU hospitals twice. Patients were the target respondents of the study. Pilot research was performed with a sample size of 72 patients to assess the components’ exploratory factor analysis (EFA). These seventy-two replies were included to the final sample size. The sampling strategy used in the current investigation was SRS.

The sample size of the quantitative research is one of the most important components [54]. Faul et al. [57] defined that G∗Power analysis programme is a reliable tool for determining the appropriate sample size for research study. The study derived sample size through G∗Power analysis. As a result, three (3) number of predictors were included in the study and the sample size was measured. Likewise, noncentrality parameter was (λ = 21.911). It is essential that “Critical F″ should be calculated for sample size selection, and it was 2.432 with numerator df (2). Denominator df was calculated (df = 467). It calculated effect size of f square which was (effect size = 0.04). For instance, power was measured, and it was (1-β err prob = 0.95). These statistical calculations derive actual power (0.950). As result, the probability err was measured for the testing hypothesis result based on (∝ = 0.05). The sample size of the research was (n = 469) from the total population.

## 7 Data analysis

The researcher used descriptive and inferential statistics to evaluate the data, using SPSS and the Structural Equation Modeling (SEM) (AMOS Version-21) tools. Several authors found that SEM (AMOS) forecasts measurement and structural models for the highly complex theoretical explanation [58,59]. Researchers tested predictive hypotheses with the help of SEM and measured the prediction between exogenous, endogenous, and intervening constructs. Descriptive statistics were used to ascertain the average and variability via the mean and standard deviation. Mediation analysis was used to comprehend the direct, indirect, and overall impact relationship among the dimensions examined in the investigation. The research used a survey procedure and collected data using modified constructs. Healthcare service quality construct was measured with 20-items (e.g., reliability, responsiveness and empathy have 13-items, tangibility 4-items and assurance 3-items). Moreover, the revisit intention scale was used of the Mohd Isa et al. [60] with 5-items. Lastly, patient satisfaction level was measured with 8-items, which was developed by Ware Jr and Hays [61]. All these responses were modified according to Urban BHU-level hospital facilities with a 5-point Likert scale. The demographic variables were controlled through quasi-experimental survey-based research, and these were purely controlled via the statistical-based method.

## 8 Reliability and validity via measurement model

The reliability and validity assessments indicated that the results were more suitable for generalization. The research constructs are defined in the methodology section of the paper. The researchers have quantified the EFA for better understand the factors sub-orders of the study, as shown in Table 1.

**Table 1:** Cross Loading and Factorization (n = 72).

Items	Loading	Cumulative %	KMO	Alpha Level
Patient Satisfaction		730.813	0.7618	0.802
PS1	0.731			
PS2	0.780			
PS3	0.771			
PS4	0.791			
PS5	0.701			
PS6	0.871			
PS7	0.801			
PS8	0.820			
Reliability, Responsiveness, Empathy		68.842	0.900	0.872
RRE1	0.813			
RRE2	0.823			
RRE3	0.848			
RRE4	0.852			
RRE5	0.871			
RRE6	0.843			
RRE7	0.861			
RRE8	0.873			
RRE9	0.891			
RRE10	0.943			
RRE11	0.919			
RRE12	0.834			
RRE13	0.862			
Tangibility		79.132	0.713	0.832
T1	0.808			
T2	0.837			
T3	0.841			
T4	0.765			
Assurance		72.139	0.821	0.882
A1	0.731			
A2	0.765			
A3	0.767			
Revisit Intention		75.400	0.808	0.836
RI1	0.894			
RI2	0.824			
RI3	0.835			
RI4	0.864			
RI5	0.823			

## 9 Relationship and measurement

The study revealed that healthcare service quality has a positive association with revisit intention. For example, healthcare service quality and revisit intention have a positive and direct correlation with patient satisfaction from the healthcare facilities. On the other hand, the average variance extracted (AVE) was measured, and the result was significant. Composite reliability (CR) and discriminant validity were measured to know the construct's validity and reliability. Moreover, mean, skewness, kurtosis, and standard deviation were statistically found to be significant for every variable (see Table 2).

**Table 2:** Correlation, validity and descriptive statistics (n = 469).

Variables	AVE	CR	1	2	3	4	5
1. Reliability, Responsiveness, Empathy	0.58	0.79	(0.80)				
2. Tangibility	0.54	0.82	0.621∗∗	(0.78)			
3. Assurance	0.53	0.87	0.531∗∗	0.631∗∗	(0.71)		
4. Revisit Intention	0.50	0.81	0.531∗∗	0.511∗∗	0.647∗∗	(0.81)	
5. Patient Satisfaction	0.56	0.80	0.741∗∗∗	0.633∗∗	0.521∗∗	0.543∗∗	(0.74)
Mean			3.179	3.321	2.678	3.800	3.931
S.D.			2.123	2.341	1.321	1.459	1.532
Skewness			0.180	0.134	0.102	0.024	0.054
Kurtosis			0.627	0.311	0.143	0.102	0.032

Note: ∗p < .05, ∗∗p < .01, ∗∗∗p < .001. “Discriminant validity is shown in bracket parallel to correlation value *Source: Survey, 2024*.

## 10 Measurement and structural analysis

**Fig. 2:**
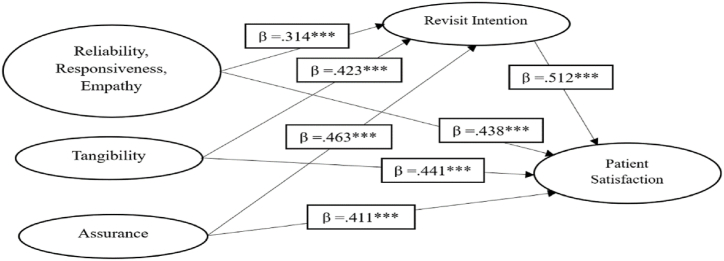
Structural model for patient satisfaction.

Source: Survey, 2024

Confirmatory measurement factor analysis was applied in this study to examine all indicators predictive association and portrayed liner relationship for each path. The study revealed that the model exhibits a substantial level of construct validity and predictability for each measurement proposition. The inclusion of these proposed variables determines that there is statistical significance among variables of the model, as seen in Fig. 2.

As such, the path diagram illustrates the theoretical justification of the relationships between causes and effects across many constructions, resulting in numerical outputs (ratio and percentages). In their study, Hair et al. [59] emphasized that establishing causal connections between predictors and outcomes is a fundamental component of path analysis. In addition, the SEM technique was developed and used to evaluate the correlation between reliability, responsiveness, empathy, tangibility, and assurance (health quality services) revisit intention and patient satisfaction. Table 3 presents the model fitness of the basic model and the numerical values of the model fit.

**Table 3:** Fit Indices for outcome (Patient Satisfaction) (n = 469).

Model	*χ*^*2*^*df*	*χ*^*2*^*/df*	*GFI*	*CFI*	*NNFI*	*RMSEA*	*SRMR*
Initial Model	11.279	9.434	0.782	0.730	0.740	0.210	0.231
Model Fit	4.147	3.435	0.911	0.854	0.873	0.071	0.083
Δχ^2^	7.132						

Note: N = 469, “All the changes in chi square values are computed relative to model, χ^2^ > 0.05, GFI = Goodness of fit index, CFI = Comparative fit index, NNFI (TLI) = Non-normed fit index, RMSEA = Root mean square error of approximation, SRMR = Standardized root mean square, Δχ^2^ = Chi square change” *Source: Survey, 2024*.

The study measures two models for the actual prediction of three exogenous, one intervening, and one endogenous construct. Such as model one was the initial model and model two was the model fit. Model one had no satisfactory results, and it was further modified through the addition and subtraction of control variables and drawing some important paths. For instance, Tomás et al. [62] described that covariance-based surveys could be significant if they draw few legitimate paths between exogenous and control factors. The study of Rauf et al. [27] applied SEM and AMOS to exogenous and endogenous factors and modified the initial model with the help of one control and two more covariance paths to improve the significance of the model fitness. Likewise, Mahmood et al. [63] modified the initial model for accurate results of the model fitness. Byrne [64] justified that covariance errors are more reliable if the difference between the initial model and model fit should be greater (Δχ2 = 4.0) after the modification process. In the second stage of analysis, the “Chi-square Chang” value was greater than 4.0, which was a good announcement for the model fit indexes. Besides, the “Δχ2” was resultantly found with the approximation of (Δχ2 = 7.132), and it is best fit for the modification process, as well as the assumption of SEM was approved. As a result, the model fit statistics are portrayed in Fig. 2 and Table 3. On the other hand, absolute and relative fitness were interpreted in Table 3, and the fitness values were maintained based on GFI, CFI, NNFI, and RMSEA. The result indicated that the model fit was perfect , and the values of the model fit were significant for every path. Similarly, GFI = 0.911, CFI = 0.854, and NNFI = 0.873 values were best fitted in the model fit indices process. Model fit results revealed that RMSEA = 0.071 and SRMR = 0.083 values were significant and best fitted for the overall model. Thus, the study indicated that goodness of fit ((χ2/df = 4.147) values were significant during the predictive analysis among constructs. In conclusion, the saturated model proved that revisit intention mediated between health quality services and patient satisfaction at the urban BHU level. The saturated model has best-fit indices, and modification was not required because the result fulfilled the criteria of model fitness (see Table 3). The study measured direct effects in the form of standardized estimation for patient satisfaction.

The study accepted that health service quality and revisit intention have a positive effect on patient satisfaction levels. Like, revisit intention mediates between service quality and patient satisfaction level. The study proved that health service quality directly and positively influences patient satisfaction levels. Whenever service quality increases, it instantly improves patient satisfaction in healthcare institutions. The findings portrayed that health service quality is the best factor for patient satisfaction at the BHU level at urban localities (reliability, responsiveness, empathy β = 0.438, tangibility β = 0.421, and assurance β = 0.411). As a result, the model portrayed that R^2^ brought 31 percent change in the whole model, which was significant 100×.312=31%. It means that the health service quality of urban BHU hospitals increases patient satisfaction levels by 31 percent (see Table 4).

**Table 4:** Direct effects of beta estimates during patient satisfaction (n = 469).

Factors	Revisit Intention		Patient Satisfaction	
	β	S.E	β	S.E
Reliability, Responsiveness, Empathy	0.314∗∗∗	0.050	0.438∗∗∗	0.056
Tangibility	0.423∗∗∗	0.052	0.441∗∗∗	0.057
Assurance	0.463∗∗∗	0.058	0.411∗∗∗	0.053
R^2^	0.312			

The results showed that revisit intention indirectly affects patient satisfaction in the context of BHU healthcare facilities hospitals. The study proposed that revisit intention mediates between service quality and patient satisfaction. The findings revealed that revisit intention indirectly affects patient satisfaction (revisit intention β = 0.512). The R^2^ change was statistically measured, and the intervening model was estimated to predict the overall variation up to 100×.433=43%. As a result, revisit intention can be improved due to patient satisfaction among urbanized respondents at the BHU level (see Table 5). In a nutshell, the research paper tested all seven hypotheses to conclude the result and follow Table 6.

**Table 5:** Indirect effects of beta estimates during patient satisfaction (n = 469).

Factors	Patient Satisfaction		
	β	S.E	CR
Reliability, Responsiveness, Empathy	–	–	–
Tangibility	–	–	–
Assurance	–	–	–
Revisit Intention	0.512∗∗∗	059	9.887
R^2^	0.433

**Table 6:** Paths and hypotheses testing for patients’ satisfaction (n = 469).

Hypotheses	Paths	Variables	Estimate	S.E.	C.R.	P	Label
Revisit Intention	<---	Reliability, Responsiveness, Empathy	0.314	0.050	6.935	∗∗∗	Sig
Revisit Intention	<---	Tangibility	0.423	0.052	7.626	∗∗∗	Sig
Revisit Intention	<---	Assurance	0.463	0.058	9.642	∗∗∗	Sig
Patient Satisfaction	<---	Revisit Intention	0.512	0.059	9.887	∗∗∗	Sig
Patient Satisfaction	<---	Reliability, Responsiveness, Empathy	0.438	0.056	8.342	∗∗∗	Sig
Patient Satisfaction	<---	Tangibility	0.441	0.057	8.427	∗∗∗	Sig
Patient Satisfaction	<---	Assurance	0.411	0.053	7.450	∗∗∗	Sig

The model fitness showed that seven proposed hypotheses were significantly accepted. Lastly, the finding concluded that improved health quality services increase patient satisfaction levels and patient revisit intention was enthusiastic toward urban BHU hospitals (see Table 6).

## 11 Discussion and conclusion

In the discussion and conclusion section, we reveal complex dynamics among healthcare service quality, revisit intention and patient satisfaction within TPB framework. The current study analysis reveals insights into how subjective norms, attitude and behavioral control influence patients’ attitude. It also addresses the importance of tangible factors of service quality, subjective perceptions and social influences that collectively shape patient experience. Further, the analysis was regulated, and therefore, the outcomes were validated for basic health units in the urban areas. The results reveal that patient satisfaction level likely improve health service quality and ultimately energize revisit intention. This innovative prediction can bring 43 % percent changes in patient satisfaction regarding BHU hospitals in urban areas. Thus; the first objective of the study is achieved. Our study findings are similar with Lai et al. [40] who proclaimed that service quality positively effect on patient satisfaction. However, this study extends the findings of Smith et al. [65] to urban areas, whereas their study was limited to rural healthcare. Our study revealed that urban healthcare institutions and inconsistency regarding healthcare facilities decreased patient satisfaction. This finding is aligned with the Asar and Abdullah [66] which validate our results and emphasize the need to regulate healthcare service in urban areas.

In conclusion, it is found that good healthcare service quality improves patients' satisfaction and it considered highest achievement for urban health units. Similarly, it also found that patient satisfaction is influenced by personal expectations in the form of service quality and continuous revisits to the same hospital for treatment which are consistent with Rahman and Suyoto [20] study. Thus; second objective of the study achieved. The predisposition of revisit intention was measured regarding health service quality and predicts patients' behavioural intentions for attaining healthcare services. The current study revealed that the revisit intention of the patients was improved due to satisfaction level. It also seen that revisiting intention directly and indirectly influences patients’ satisfaction when knowledge and learning involved in the service quality of the healthcare centres. Thu; third objective of the study is achieved.

In light of healthcare service quality, patient satisfaction could attract their intention to visit different medical healthcare hospitals in metropolitan vicinity [17]. The contemporary study recommended that patient satisfaction is an important linear theoretical foundation to revisit again in the same hospital [6]. Finally, the predictive analysis leads to some useful conclusions, the most important of which is adding revisit intention factors to the BHU level for acquiring satisfaction levels among patients, which can be possible with this new model. Some of the broad conclusions drawn from the results, such as revisit intention, intervened positively between service quality and patient satisfaction in the BHU hospitals. Since health service quality can have serious consequences, it is imperative to better understand the phenomenon of revisiting intentions in different healthcare institutions. The validation process confirmed that the theory of planned behaviour is the framework to produce behavioural intention among patients, and they reviewed again to good service quality hospitals. The targeted intervention in healthcare services can effectively cultivate an environment conducive to positive patient experiences and foster loyalty through increased revisit intention.

## THEORATICAL contributions and practical implications

The present study has notable contributions and implications based on current study construct and their implementation mechanism. The current study provides valuable insights to the theory of planned behaviour and its application in patient behavioral intention in BHUs context. This study also contributes to the extent literature by measuring rarely investigated role of behavioral intention between service quality and patient satisfaction. Particularly, current study findings reinforce the relevance and understanding of theory of planned behaviour in-patient decision-making process and emphasize its use in predicting revisit intention. This theoretical validation will increase our understanding regarding patient's behaviour and also provide theoretical framework to the future researchers who seeking to explicate patient satisfaction in healthcare context. Additionally, current study's practical implications allow healthcare service provider to focus on improving communication strategy to enhance patient satisfaction, which influence positive attitude regarding healthcare service at BHUs. The hospital administrators should initiate tailored program for healthcare practitioners to increase patient compliance and promote culture aptitude in urban and rural areas. The hospital administrators should take initiative to make service more convenient and accessible through efficient appointment system which may strengthen patients behavioral intention.

## Limitations and future Directions

The present study has some limitations that offer opportunities for potential researchers. First, current study depends on self-reported data from patients which may have response bias regarding healthcare services and patient satisfaction. Secondly, current study may not comprehensively capture multifaced nature of satisfaction, as certain aspects or dimensions of service quality in basic health units may not adequately assessed. Thirdly, cross-sectional study design may preclude the casual relationship among variables over time, vice versa longitudinal study may elucidate temporal dynamics effectively. Additionally, mixed-method approaches could provide comprehensive insight in term of contextual factors influencing behavioral intention within basic health units. Finally, comparative studies across different region and healthcare facilities may provide variation in level of service quality and patient satisfaction, informing intervention to improve healthcare delivery.

## Ethics statement

No animal studies are presented in this manuscript. “This study involving human participants was reviewed and approved by The TIMES Institute, Office of Research, Innovation and Commercialization (ORIC), Multan, Pakistan Ref No (TI/ORIC/2023/86). The participants provided their oral consent to participate in this study.”

## Data availability statement

Data will be made available on reasonable request.

## Funding

The authors received no direct funding for this research.

## CRediT authorship contribution statement

**Abdul Rauf:** Writing – review & editing, Writing – original draft, Methodology, Conceptualization. **Norhilmi Muhammad:** Visualization, Validation, Supervision, Conceptualization. **Hamid Mahmood:** Writing – review & editing, Visualization, Software, Methodology, Formal analysis. **Yuen Yee Yen:** Software, Methodology, Data curation.

## Declaration of competing interest

The authors declare that they have no known competing financial interests or personal relationships that could have appeared to influence the work reported in this paper.
